# Unusual progression of renal cell carcinoma with carcinomatosis peritoneii and Krukenberg tumour and alopecia with sunitinib therapy in young female

**DOI:** 10.1186/s12957-018-1328-3

**Published:** 2018-02-06

**Authors:** Manoj Pandey, Mahendran Ramasamy, Mridula Shukla

**Affiliations:** 1 Department of Surgical Oncology, Institute of Medical Sciences, Banaras Hindu University, Varanasi, 221005 India; 2 Lab Head, SRL Religere, Varanasi, 221005 India

## Abstract

**Background:**

Sunitinib is a multiple receptor tyrosine kinase inhibitor (TKI) used for the treatment of renal cell carcinoma (RCC). It increases the median survival considerably with minimum side effects. Alopecia is one of the rare side effects. Metastasis to the ovary is also rare. We report a case of RCC metastasizing to the ovary developing alopecia early on starting sunitinib.

**Case presentation:**

A 22-year-old hypothyroid girl underwent right radical nephrectomy for T_2_N_0_ RCC. Histopathology was clear cell carcinoma. Six months later, she presented with right iliac fossa pain, imaging revealed metastasis to the ileocolic junction and the ovary, an exploratory laparotomy was carried out and, after debulking, the patient was started on sunitinib. Four weeks after the start of the  treatment, she developed alopecia. She was continued with sunitinib therapy till progression.

**Conclusions:**

The present case shows a rare metastasis to the ovary and early onset of rare adverse event of alopecia on starting sunitinib therapy. In the presence of confounding factors like hypothyroidism and dandruff, establishing this as an adverse reaction of sunitinib is difficult. This case had a unique metastatic spread with involvement of the bowel, ovary and peritoneal carcinomatosis. Use of adjuvant TKI's after resection of primary tumour in nonmetastatic setting may reduce metastatic rates and increase progression-free survival.

## Background

Sunitinib is a novel multitargeted tyrosine kinase inhibitor of vascular endothelial growth factor receptors, platelet-derived growth factor receptors and other RTKs with antitumour and antiangiogenic activity [1]. It is accepted worldwide for the treatment of metastatic renal cell carcinoma (mRCC) and imatinib-resistant gastrointestinal stromal tumours (GIST) [2]. Long-term survival in patients receiving sunitinib for mRCC is > 2 years with a follow-up of > 6 years [3]. Treatment-related adverse effects (TRAEs) following long-term follow-up included decreased appetite, diarrhoea, dysgeusia, dyspepsia, fatigue, hypertension, mucosal inflammation, nausea, stomatitis and hypothyroidism [4]. Though metastasis is common, metastasis to the ovary is rare with only 34 cases been reported till date [5, 6]. We present a case of alopecia as a TRAE of sunitinib in a patient of mRCC to ovary carcinomatosis peritoneii in the early phase of treatment.

## Case presentation

**Fig. 1:**
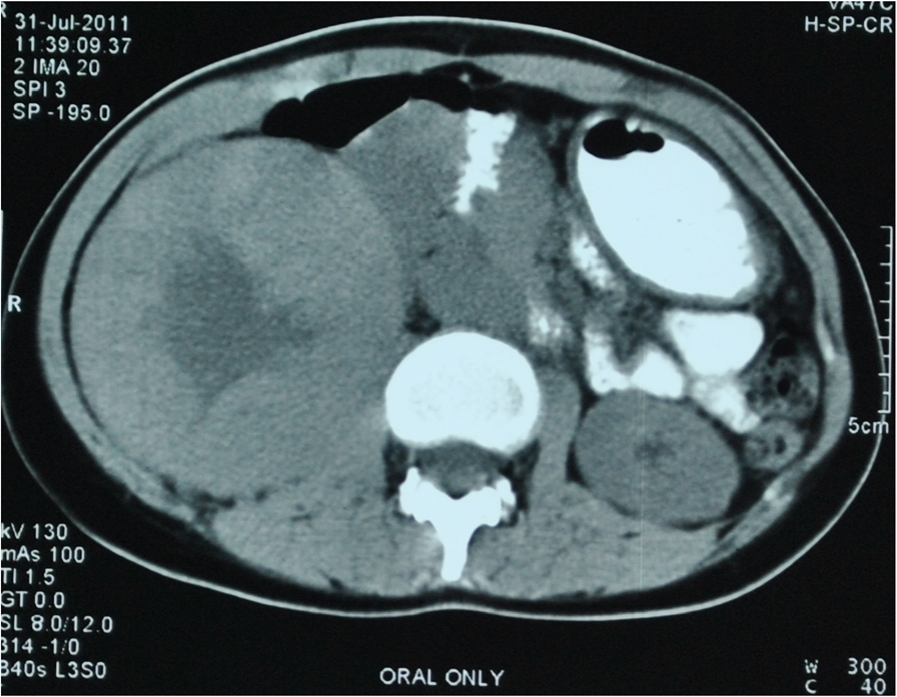
Computed tomogram showing large mass replacing almost whole of the right kidney with a functioning left kidney

**Fig. 2:**
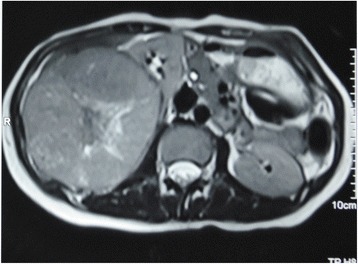
MR image showing mass in the right kidney

**Fig. 3:**
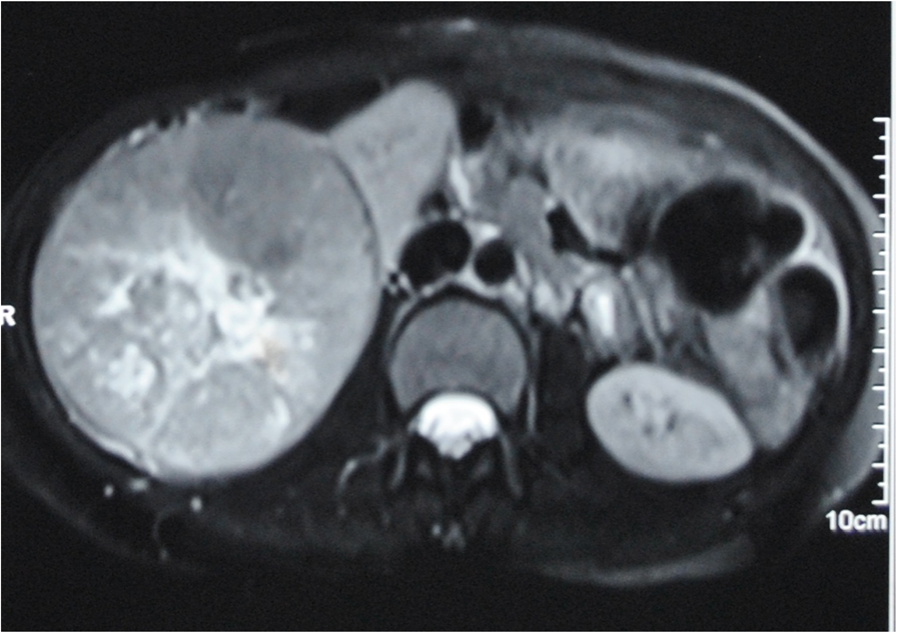
T2-weighted MR image showing a mass replacing whole of the kidney with no extracapsular invasion

**Fig. 4:**
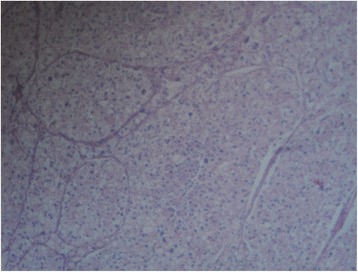
Photomicrograph showing clear cell carcinoma of the kidney (haematoxylin and eosin × 40)

**Fig. 5:**
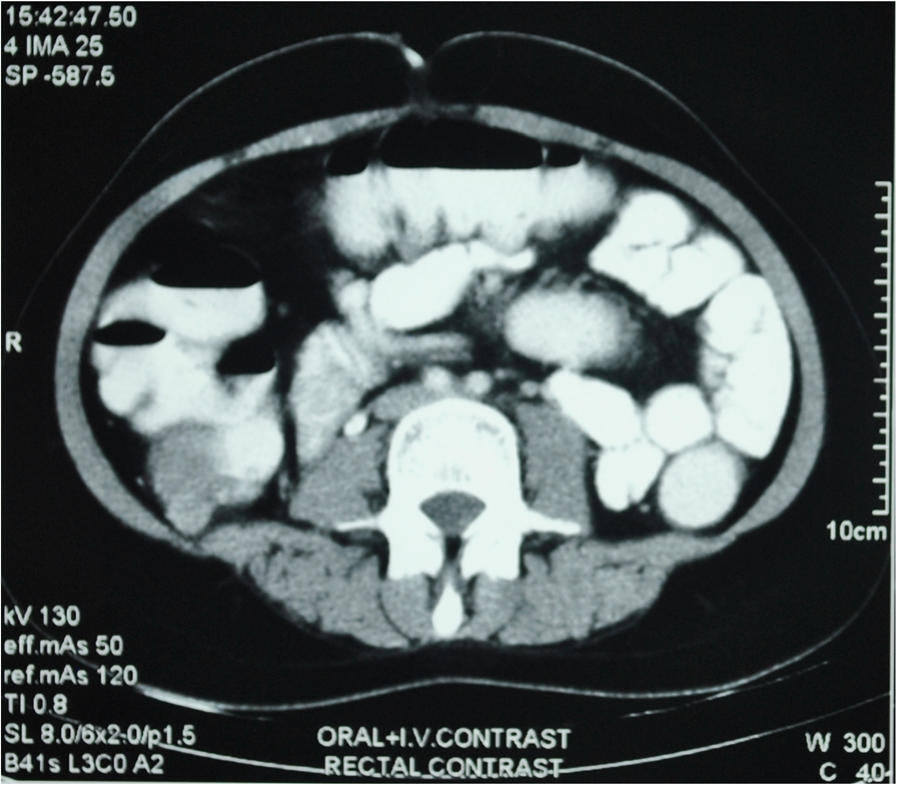
Computed tomography image showing mass at the ileocolic junction; no recurrence is seen in the renal bed

**Fig. 6:**
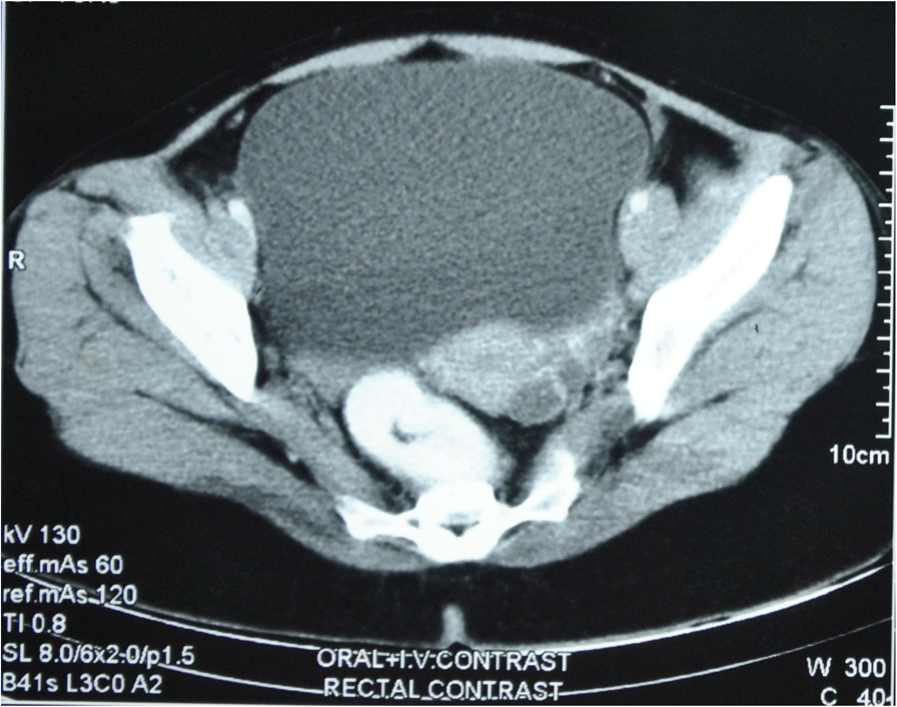
Computed tomography image showing collection in the uterine cavity with lesion in the left adnexa

**Fig. 7:**
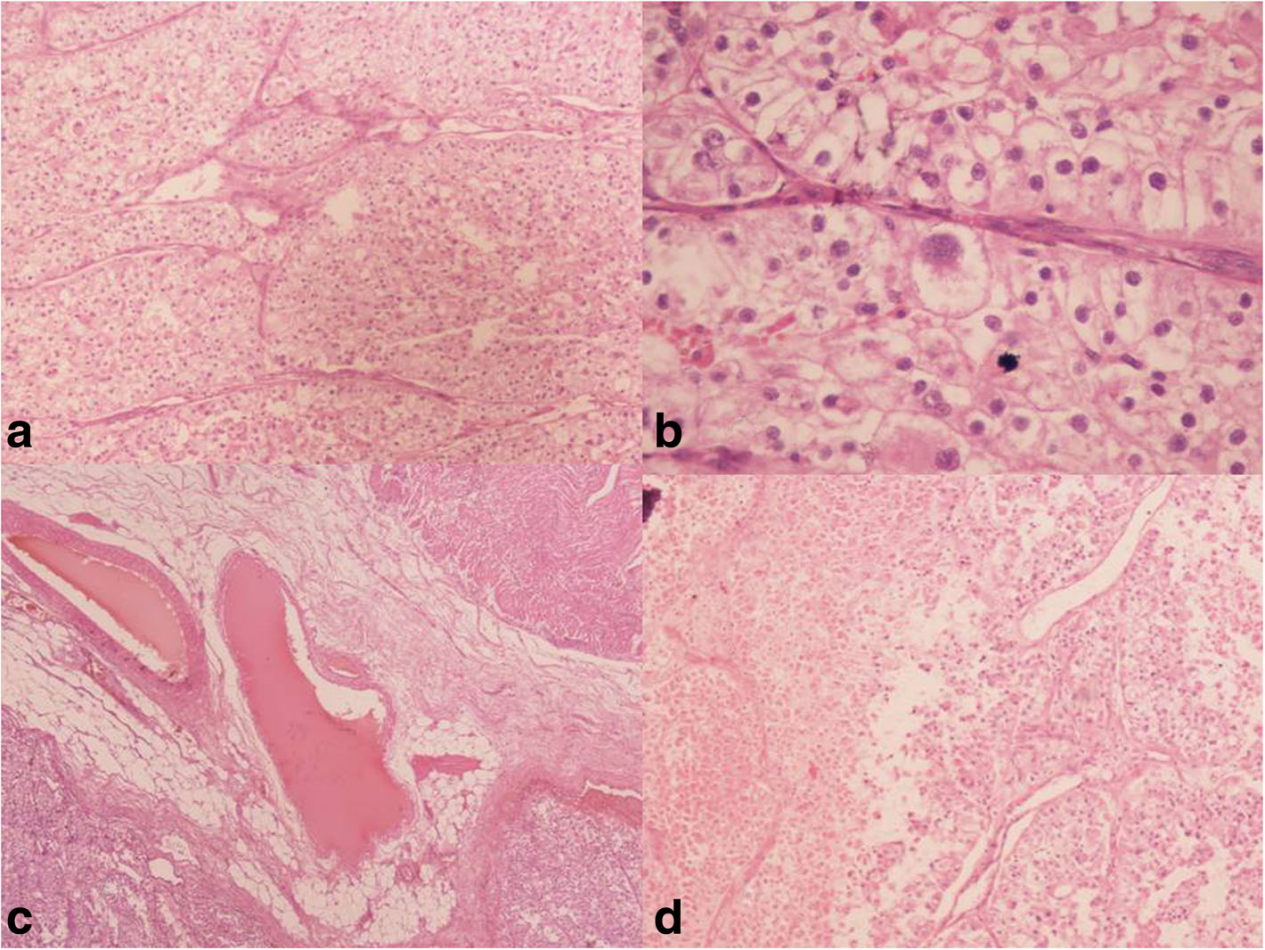
Photomicrograph of the recurrent tumour. **a** Low power view showing nests of clear cells (H&E × 40). **b** High power view showing clear cell deposits (H&E × 400). **c** Clear cell deposits in the mesentry with smooth muscle wall on top right and tumour on bottom left (H&E × 40). **d** Low power view showing necrosis on the left and tumour on the right (H&E × 40)

A 22-year-old hypothyroid girl presented with right renal mass (Figs 1, 2 and 3). A metastatic workup was negative. Haematological parameters were normal; however, the patient was found to be hypothyroid with T3 of 93.6, T4 of 4.2 and TSH of 11. With a diagnosis of T_2_N_0_, RCC, the patient underwent right radical nephrectomy after correction of her hypothyroid status. At the time of surgery, the thyroid hormone status was T3 of 112, T4 of 14 and TSH of 0.5. Histopathological examination revealed a clear cell carcinoma Fuhrman nuclear grade 4, of the right kidney pT_2a_N_0_ (Fig. 4). Gerota’s fascia was intact, and all resection margins including the renal vein and Gerota fascia were negative. The lymphovascular invasion was present, and the tumour necrosis was also present. Patient made and uneventful recovery and was discharged on the 5th postoperative day. Six months later, she presented with pain in the abdomen, and examination revealed a right iliac fossa mass. Her thyroid status was again found to be deranged with a T3 of 67, T4 of 5.9 and TSH of 16. Imaging revealed metastasis at the ileocolic junction and the ovary (Figs. 5 and 6). After correction of thyroid status (T3–76, T4–11, TSH–2.8), she underwent an exploratory laparotomy; on laparotomy, multiple peritoneal nodules were found with 4-cm lesion at the ileocolic junction, 3-cm lesion in the right ovary and 1-cm lesion in the left ovary. A right hemicolectomy, and a right oophorectomy with excision of all peritoneal nodules, was carried out. The histopathology was consistent with metastatic clear cell carcinoma; one of the dissected pericolic nodes was also positive (Fig. 7). The metastasis was present on the surface of the resected organs and peritoneum with mucosa of the colon being normal; a good amount of tumour necrosis was also present. She was started on sunitinib therapy. Four weeks after starting the therapy, she developed alopecia. She was referred to her endocrinologist as she was hypothyroid on supplementation, who found the thyroid function to be within normal limits. She was then referred to a dermatologist who found that patient has dandruff and started her on an anti-dandruff treatment. The dandruff got controlled, but the hair fall continued as it was earlier. A diagnosis of sunitinib-induced alopecia was made; this was discussed with the patient, and she made a decision to continue on sunitinib therapy. Eight months after the treatment, she had a progression of disease and ascites and she was offered to shift to mechanistic target of rapamycin (mTOR) inhibitors which she refused. After discussions, she was started on sorafanib 400 mg OD. Two months after starting on sorafanib therapy, the patient died of progressive disease at home.

## Discussion

Alopecia is a little discussed side effect of tyrosine kinase inhibitors (TKIs) and is usually milder than that in chemotherapy. A PubMed search carried out in November 2016 using the term (“sunitinib” [Supplementary Concept] OR “sunitinib” [All Fields]) AND (“alopecia” [MeSH Terms] OR “alopecia” [All Fields]) revealed only 13 articles [7–19] of which only five articles [7–11] analysed the adverse effect of sunitinib. Changal et al. reported incidence of alopecia in 13% of patients treated with sunitinib for mRCC [7]. Another study from China shows alopecia in 34% of patients [8], while results from other studies by Chan and Lee show alopecia in 6 and 5% of patients respectively [9, 10]. Pooled analysis of published studies retrieved from various databases by Rosenbaum shows alopecia in 6% of patients [11]. However, the studies that compared the adverse effect of TKIs show alopecia to be more common in sorafenib (26%)- and pazopanib (11%)-treated patients when compared to sunitinib (6%) [12–14]. Recent pooled analysis of 5739 patients from various trials which analysed long-term TRAE of sunitinib concluded that prolonged sunitinib was not associated with new types or increased severity of TRAEs. Alopecia was not included as an adverse effect in that trail [18]. Our patient developed alopecia within 4 weeks of initiation of sunitinib therapy. Her thyroid status at that time was normal. No other known confounding factor was found. Dermatological and endocrine consultations were done; that too suggested sunitinib as the probable cause of alopecia.

Almost one third of the patients have metastasis at the time of presentation, while others develop metastasis during follow-up. Metastatic spread to the lung, liver, bones and lymph nodes is common in clear cell carcinoma of the kidney. Peritoneal carcinomatosis and metastasis to the bowel and ovary are very rare with only 34 cases of ovarian metastasis being published in PubMed-indexed literature till November 2016 [5, 6]. Another PubMed search with term (“carcinoma, renal cell” [MeSH Terms] OR (“carcinoma” [All Fields] AND “renal” [All Fields] AND “cell” [All Fields]) OR “renal cell carcinoma” [All Fields] OR (“renal” [All Fields] AND “cell” [All Fields] AND “carcinoma” [All Fields])) AND ((“carcinoma” [MeSH Terms] OR “carcinoma” [All Fields] OR “carcinomatosis” [All Fields]) AND peritonei [All Fields]) was carried out on December 16, 2017, revealed four articles, and none of them were on renal cell carcinoma. However, hand-searching the literature identified 19 instances where peritoneal dissemination had been reported. These can be classified into three subcategories, i.e. first as presentation with disseminated peritoneal disease, second as recurrence after initial nephrectomy [20] and third, most common nowadays, as port site and omental mets after the laparoscopic excision of renal masses. Stavropoulos et al. reported a case who presented with peritoneal metastasis and noted that the incidence of peritoneal metastasis in renal cell carcinoma is about 1% at autopsy [21]. The first port site metastasis was probably reported for the first time in 2006 by Dhobada et al. [22], while recurrence in peritoneum was reported in 1990 [23]. It is now clear that 1–3% of all cases of renal cell carcinoma develops peritoneal metastasis and the literature review suggest that transcoelomic spread may be one of the ways RCC disseminates, besides hematogenous and lymphatic dissemination. Though theoretically, peritoneal implantation at the time of surgery could be a possibility and may be a reason for port site metastasis, in tumours where Gerota’s fascia has been intact and tumour has been confined to the kidney, transcoelomic spread is a possibility. The median survival of patients with metastatic disease is about 4 months while it increases with the use of TKIs and mTOR inhibitors, and the survival depends on the grade of the tumour and prognostic grading criteria that are proposed.

## Conclusions

Alopecia is a dermatological side effect that can also be seen with sunitinib therapy. Though not so rare, this is a little known adverse event of this therapy. In presence of hypothyroidism and dandruff, establishing the cause of alopecia to sunitinib therapy is only by exclusion and correction of the two. Further ovarian metastasis is also rare with only 34 cases been reported till date. This case had unique metastatic spread with involvement of the bowel, ovary and peritoneal carcinomatosis. Use of adjuvant TKIs after resection of primary tumour in nonmetastatic setting may reduce metastatic rates and increase progression-free survival.
